# Urban pearl millet farmers’ perceptions of climate change and adaptation strategies in Niamey commune V, the Sahel

**DOI:** 10.1038/s41598-025-14952-7

**Published:** 2025-09-29

**Authors:** Gnanki Mariam Lafia N‘ Gobi, Soulé Moussa, Hamidou Falalou, Pamphile Degla

**Affiliations:** 1 West African Centre for Sustainable Rural Transformation, University Abdou-Moumouni , Niamey, Niger; 2 University Dan Dicko Dankoulodo, Maradi, Niger; 3 International Crops Research Institute For The Semid-Arid Tropics (ICRISAT) Sahelian Centre, Niamey, Niger; 4 Faculty of Agronomy, University of Parakou, Parakou, Bénin

**Keywords:** Climate change, Urban farmers, Pearl millet, Adaptation strategies, CSA, Niger, Climate-change adaptation, Climate-change impacts, Climate-change mitigation, Climate-change policy, Sustainability

## Abstract

Climate variability in the Niamey region presents a dual-faceted challenge. Yet most studies focus on broader farming systems. This study investigated the climate change perceptions and adaptation strategies developed by urban pearl millet farmers in Niamey Commune V, an area often overlooked in agricultural studies. Using snowball sampling, data were collected on socio-demographic and socio-economic characteristics, climate change perception, and adaptation strategies through structured interviews, focus group discussions, and analysis of 30 years of rainfall data (1991–2020). A sample of 150 pearl millet farmers aged at least 40 years was surveyed, and key informant interviews were conducted with the Agricultural Extension Unit. Descriptive statistics, statistical tests, and multinomial logistic regression were used to identify the determinants of adaptation strategy adoption. Findings revealed a high level of climate awareness among farmers over the past 30 years, which led to the adoption of both local and extension-based adaptation measures. Significant disruptions in rainfall were noted. Farmers primarily used soil fertility regeneration techniques, crop diversification, crop defense, improved seeds, organic fertilizers, adjusted planting calendars, water conservation techniques, and prayers or rituals to cope with these changes. Local practices aimed at improving productivity and climate adaptation, while extension-derived practices emphasized the synergy between productivity, adaptation, and mitigation. This research addresses a critical knowledge gap in how urban pearl millet farmers perceive and respond to climate change impacts. The study’s findings are significant for urban agriculture policy, underscoring the need for timely climate information, effective extension services, and the integration of adaptive agricultural practices into urban planning. These steps are crucial for enhancing resilience and fostering sustainable development in urban agriculture.

## Introduction

Climate change is increasingly recognized as a critical global development issue affecting many sectors in the global economy. It is widely recognized as one of the most serious threats to sustainable development (Akinnagbe and Irohibe, 2015). According to the IPCC Fifth Assessment Report (IPCC, 2022) , the agriculture, forestry, and other land use (AFOLU) sector is responsible for approximately 22% of global greenhouse gas (GHG) emissions. These emissions primarily result from methane produced by livestock and nitrous oxide released from soils due to fertilizer application. While this sector contributes significantly to global warming, it is also among the most vulnerable to its impacts, particularly in regions with limited adaptive capacity. For instance, it has been estimated that between 2006 and 2016, agriculture was affected by more than 26 percent of all damage and losses caused by climate-related disasters and up to 83 percent of all damage and losses caused by drought in developing countries (FAO,^1^). In Sub-Saharan West Africa, climate events have become more intense and frequent than ever, leading to severe changes in the patterns, timing, and precipitation levels^2^. The region’s susceptibility to climate change is exacerbated by its heavy reliance on natural resources, rainfed agriculture, and the environment for sustaining food security and livelihoods. Consequently, this results in persistent humanitarian crises characterized by recurring droughts, floods, food shortages, epidemics, and violent conflicts (USAID, 2017). Actually, climate change has been impacting several sectors of agriculture in West Africa in several ways affecting both food and non-food crops either through increasing temperature, flash flooding, or severe droughts (GIZ, 2018).

Niger, situated in the heart of the Sahel, is particularly vulnerable due to its heavy reliance on rainfed agriculture (Haut Commissariat de l’Initiative 3N, 2012) and its rapidly growing population. Each year, over 30,000 houses, 20,000 hectares of agricultural land and more than 10,000 heads of livestock are affected by the recurrent floods (Bori, 2021). These climatic shifts threaten the productivity and sustainability of staple crops such as millet, which accounts for over 65% of the sown area of most of the cropping systems) (Kadri et al.^3^), which plays a central role in food security and rural livelihoods.

In recent years, urban and peri-urban agriculture has become increasingly important in Niger where it serves as the primary income source for approximately 50% of households^4^, especially in the capital city of Niamey. According to the National Institute of Statistics of Niger (Institut National de la Statistique du Niger,^5^), more than 40% of households in Commune V are engaged in some form of urban farming, either for subsistence or income generation. Among its five communes, the Commune V of Niamey stands out for its high concentration of urban farmers cultivating millet (41,046 over 120,643 tons from 2010 to 2017) (INS,^5^) and vegetables on available plots. This could potentially be attributed to the climate resilience of the crops and farmers’ consumption patterns in the commune.

Despite this trend, over the years, most research on farmers’ perceptions or local knowledge of climate change and its impact on crop production has focused on rural areas, likely because agriculture remains the primary livelihood in those regions. While peri-urban agriculture plays a vital role in ensuring food security and livelihoods in urban settings, it has received limited scholarly attention. Existing studies have predominantly focused on urban horticulture^6^ and ruminant husbandry^7,8^,etc.) rather than on large-scale crop production.

Recent research highlights the potential of nature-based solutions, such as agroforestry, multifunctional landscapes, and crop diversification, in supporting farmer adaptation in vulnerable regions^9^,^10^. Other studies have focused on farmers’ local knowledge of various climate shocks^11^, rural farmers’ perception of climate change^12,13^), and the factors influencing these perceptions^14^. However, these studies primarily focus on rural agriculture. Given the significant role agriculture plays in ensuring food security in urban areas, particularly in the city of Niamey, it is essential to explore how urban farmers are integrated into the agricultural system and to assess the measures implemented to sustain production amid climate variability. To better understand the decision-making processes behind farmers’ adaptation strategies, this study adopts the Theory of Adaptive Behavior (TAB) developed by Eduardo Chia et al.^15^. Unlike the neoclassical production theory, which assumes fixed profit-maximization objectives, TAB acknowledges that farmers operate under evolving goals and constraints. It conceptualizes decision-making as a dynamic process shaped by context, experience, and perception, making it especially relevant for analyzing behaviors in climate-vulnerable settings. Farmers’ personal characteristics such as age, experience, education, and risk perception as well as their perceived socio-economic status and environmental vulnerability, continuously influence and reshape their goals and constraints over time. This approach provides a realistic framework to better understand how urban millet farmers in Niger adapt their strategies in response to environmental and socio-economic pressures.

This study seeks to address this gap by investigating the perceptions and adaptation strategies of pearl millet farmers in Commune V of Niamey. Gaining insights into how these farmers who frequently operate within informal systems with limited institutional support, adapt with climate variability, is essential for informing the design of inclusive and effective adaptation strategies.

## Materials and methods

### Study area

This study was conducted in the Niamey region, situated in the southwestern part of Niger, where pearl millet is the dominant crop. The selection of this area is informed by both its agro-ecological significance and its socio-economic reliance on millet farming, underscoring its relevance for a study on agricultural practices. Specifically, the study focused on Commune V, the region’s most productive millet-growing, which accounted for approximately 41,046 tons of millet between 2010 and 2017. Commune V is located on the right bank of the Niger River (Service de l’aménagement et du Territoire commune V,^16^).

Niamey is situated within the Sudano-Sahelian climatic zone, characterized by a tropical climate with a distinct dry season and pronounced temperature variations. During the hottest months (April to May), temperatures can rise to 45°C, while during the coolest period (typically in December), they may fall to approximately 15°C. The Region receives an average annual rainfall of around 600 mm, but it shows high interannual variability and is often unevenly distributed across both time and space. The topography of the Niamey region is predominantly flat, with an average elevation of 185 meters, including flood-prone zones below 182 meters and non-floodable islands. To the south, the terrain gradually rises into lateritic plateaus, and isolated hills in the area can reach altitudes above 260 meters. The region features diverse soil types: sandy soils used primarily for millet cultivation; clay-loam soils in the river valleys; lateritic soils on plateau slopes; and stony soils on upland and hilly areas (Service de l’aménagement et du Territoire commune V,^16^).

**Fig. 1:**
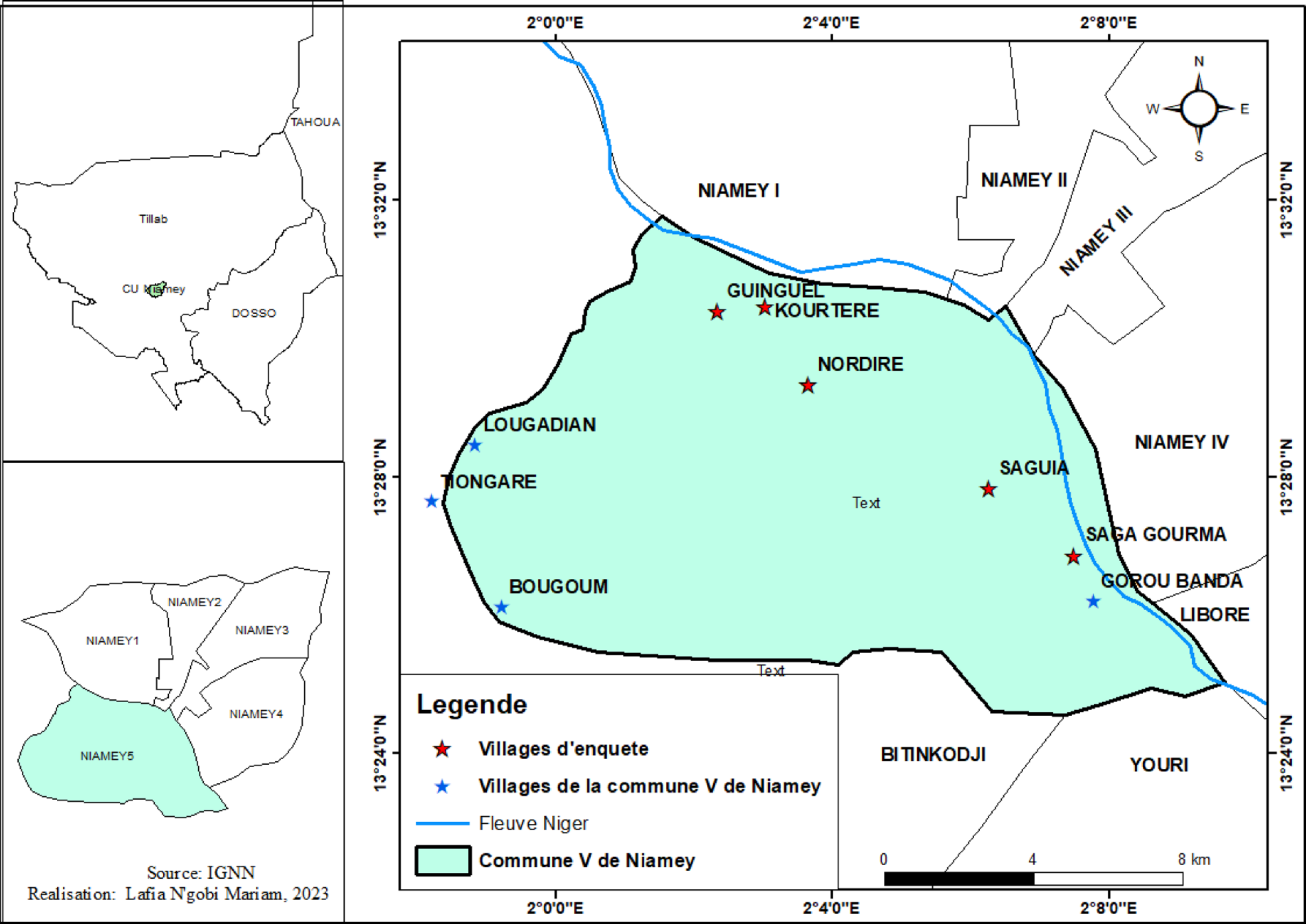
Map of study Area. Source: Author 2023

The Niamey region is bordered to the north by the Niger River and to the south, west, and east by the Kollo department. Covering an area of 40 km^2^, it hosts a population of 168,333 inhabitants and comprises 19 administrative communities, including both peri-urban and agrarian zones: Djadjiré, Karé, Karayel, Loguel, Djologou, Yawaré, Ganguel, Kossey, Kourtéré Samboro, Kourtéré Boubacar, Bougoum, Saga Gourma, Gorou Banda, Gorou Kirey, Neni Goungou, Timéré, Saguia, Nodiré and Diamowé. For this study, we selected eight communities, Saguia, Nodiré, Ganguel, Kourtéré Samboro, Saga Gourma, Kourtéré Boubacar, Loguel, and Yawaré, which represent the largest pearl millet producing villages in Commune V (Fig. 1). The selection was based on local production statistics, accessibility, and representation of diverse farming systems within the commune, ensuring both geographical and agricultural significance. All participants were adults and gave verbal informed consent prior to their participation in the research.

### Data collection methods and analysis

The objective of this study was to explore the perception of climate change among pearl millet farmers in Commune V of Niamey, Niger. Specifically, the research aimed to (1) assess pearl millet farmers’ perceptions of climate change; (2) analyze local rainfall trends over the past 30 years; (3) identify and classify the adaptation strategies adopted by farmers; and (4) evaluate the climate-smartness of these adaptation strategies.

A mixed-methods approach, combining both qualitative and quantitative techniques, was adopted to gain a comprehensive understanding of the subject. Fieldwork was conducted between August and September 2022 in the eight main millet-producing agrarian communities of Commune V.

Data collection relied on a combination of participatory diagnostic tools, informal semi-structured interviews, and formal structured surveys conducted with individual farmers and extension agents. This approach aimed to capture and analyze pearl millet farmers’ perceptions of climate change. The socio-economic data gathered included variables such as gender, age, farming experience, and interactions with extension services, along with their perceptions of climate variability and the adaptation strategies they employed. Additional socio-demographic information was collected to identify factors that might influence farmers’ perceptions. A desk review of official reports, academic articles, and relevant literature was also conducted to contextualize and support the field findings.

The study combines both quantitative and qualitative approaches. Purposive sampling was implemented using a snowball sampling strategy. Participants were selected based on a key inclusion criterion: being at least 40 years old. This age threshold was established to ensure that respondents had sufficient experience with climate change and its impacts, thereby enhancing the reliability and relevance of the information gathered. Due to the lack of an official registry of farmers in the municipality, and as local authorities were unable to provide such a list, snowball sampling allowed for the identification of eligible participants through peer referrals. This approach proved effective in reaching knowledgeable farmers and conducting in-depth interviews despite the constraints of limited data sources. Although the sample size may seem small for large-scale generalization, it is justified by field constraints and reflects the accessibility and willingness of farmers to participate in the study in an urban Sahelian context, where mobilization can be particularly challenging due to logistical, social, and environmental factors. Sample size was adopted similar to past studies in the area^17,18,12,19^.

While snowball sampling is often criticized for potential bias in quantitative research, it is commonly used in rural development studies involving hard-to-reach or specialized populations (e.g., experienced farmers) and was considered appropriate in this context^20–23^.

Hundred and fifty (150) pearl millet farmers from the eight rural and agrarian communities of Niamey Commune V participated in the study. 25 Farmers were interviewed in each of the communities.

The sample size was established using the Slovin formula (Ellen, 2019) :

$$\text{n }= \frac{N}{(1 +\text{ N}{e}^{2})}$$

where: n is the sample size, N the population size (number of agricultural households) =3631, e = margin of error= = 8 %, 1 = constate value. Using the values in Equation (1), we have sample size of 150 for this study.

The data collected were tabulated and statistically analyzed to interpret the results. Quantitative data were analyzed using SPSS version 23, STATA 17, and Excel 2022. These softwires are widely used in statistical and econometric analysis in the field of agricultural economics and social sciences. They were chosen for their flexibility, the availability of relevant functions for descriptive statistics and multinomial logistic regression, and compatibility with the data structure. Descriptive statistics were used to summarize perception patterns.

In line with previous studies that have explored farmers’ adaptation strategies using Probit or Logit models (^24,25^ , ^14^), this study adopts a binary logistic regression (logit) model to identify the socio-economic and demographic variables influencing pearl millet farmers’ responses to climate change (farmers’ choice of adaptation strategies). The choice of this model is motivated by the binary nature of the dependent variable, which reflects whether or not a farmer adopts at least one climate change adaptation strategy. The logit model estimates the probability of adaptation as a function of eight explanatory variables, including age, access to climate information, education level, gender, household size, farm size, membership in a farmer association, and contact with extension services. These variables were grounded in both literature review and conceptual frameworks related to climate change adaptation in agriculture^9,22,13,25^. 

The decision (${{\varvec{A}}}_{{\varvec{i}}}$) of a pearl millet farmer ***i*** to adopt an adaptation strategy in response to climate change is assumed to depend on a set of socio-economic and demographic characteristics. This relationship can be expressed in the general functional form:

$${{\varvec{A}}}_{{\varvec{i}}=}\mathbf{f}\boldsymbol{ }({{\varvec{z}}}_{{\varvec{i}}})$$

**Where **${{\varvec{A}}}_{{\varvec{i}}}$ represents the adaptation decision of farmer ***I***, and ${{\varvec{Z}}}_{{\varvec{i}}}$ *denotes* a set of socio-economic and demographic variables specific to the same pearl millet farmer.

The logistic regression equation is as follows:

$$\mathbf{P}(\mathbf{y}=1|\mathbf{X})=\frac{1}{1+{{\varvec{e}}}^{-({\varvec{\beta}}0+{\varvec{\beta}}1{\varvec{X}}1+{\varvec{\beta}}2{\varvec{X}}2+\dots .{\varvec{\beta}}{\varvec{n}}{\varvec{X}}{\varvec{n}})}}$$

where:

- P (y = 1|X) is the predicted probability that the dependent variable **y** is equal to 1, given the explanatory variables **X**.
- X1, X2, ..., Xn are the **n** independent variables or explanatory factors.
- $\beta$ 0, $\beta$ 1, $\beta$ 2, ..., $\beta$ n are the coefficients of the model, estimated through an optimization procedure such as maximum likelihood estimation
- *e* is the Euler number (e = 2.718...), which is a mathematical constant.

### Climate-smartness indicators for adaptation strategies

Climate Smart Agriculture (CSA) is defined as agriculture that sustainably increases productivity, enhances resilience (adaptation), reduces/removes greenhouse gas emissions (mitigation), and contributes to national food security and development objectives (FAO,^1^). CSA includes a broad range of approaches, combining traditional practices such as mulching, intercropping, conservation agriculture, and pasture and manure management with innovative strategies, programs, and policies like improved crop varieties, an advanced weather forecasting system, and agricultural risk insurance. In line with this definition, the study assessed the climate-smartness of local adaptation practices using a standardized evaluation framework.

The evaluation framework incorporates a set of suggested indicators for assessing practices in relation to the three CSA pillars (productivity, adaptation, and mitigation), including metrics such as yield, income generation, water use efficiency, and emissions intensity^26^.

The CSA assessment tool developed by the CGIAR-CCAFS program was employed to quantify the climate-smart potential of each adaptation strategy. This tool evaluates practices based on three pillars:

- Productivity: Ability to improve yield or production (feed/yield-smartness and income-smartness)
- Adaptation/resilience: water-use and soil-use efficiency, risk reduction.
- Mitigation: energy, carbon, and nitrogen smartness).

Each adaptation practice was scored on a scale from -10 (strongly negative impact) to +10 (strongly positive impact) for each indicator. Average scores were then calculated for each CSA pillar, allowing comparisons across practices. This scoring system, used in previous studies such as Andrieu et al.^27^, which employed the same tools to assess the climate-smartness of adaptation practices developed in Mali, ensures consistency and comparability in the evaluation process.

## Results and discussion

### Descriptive statistics

The pearl millet farmers surveyed were predominantly male (61.3%), with low levels of formal education: 51.3% are illiterate and 22.7% have only primary education. The majority (72.7%) are crop farmers, while 10.7% are involved in animal farming or agropastoralism. Beyond crop cultivation, 84% engaged in secondary activities such as handicrafts, petty trade, teaching, fishing, or livestock keeping, reflecting the diversification of livelihoods in response to environmental and economic pressures. The communities are largely Muslim (94%). The average age of respondents is 52 years (±10), with approximately 28 years (±2) of farming experience, and most have between 26 and 30 years of specific experience in pearl millet production. This profile suggests a mature and experienced farming population with potentially strong empirical knowledge of local climatic changes. These socio-demographic characteristics offer a relevant background for understanding the farmers’ perceptions and adaptation behaviors discussed in the following sections (Table 1).

**Table 1:** Socio-demographic characteristics of pearl millet farmers (qualitative and quantitative variables).

Qualitative variables		Frequency	Percentage (%)
Sex of the farmer	Woman	58	38.7
	Man	92	61.3
Religion	Christian	9	6.0
	Muslim	141	94.0
Level of education	Illiterate	77	51.3
	Primary	34	22.7
	High school	3	2.0
	College	14	9.3
	Technical school	1	0.7
	Coran	18	12.0
	University	2	1.3
	Apprenticeship	1	0.7
Main activities	Agriculture	109	72.7
	Breeding/agro-pastoralism	16	10.7
	Employee/worker	5	3.3
	Civil servant	5	3.3
	trade	12	8.0
	Handicraft	1	0.7
	Other	2	1.33
Do you have any secondary activity?	No	24	16.0
	Yes	126	84.0
Experience in pearl millet cropping	1-5 years	5	3.33
	6-10 years	28	18.67
	11-15 years	14	9.33
	16-20 years	28	18.67
	21-25 years	17	11.33
	26-30 years	32	21.33
	Beyond 31 years	26	17.33
Quantitative variables	Minimum	Mean (St.d)	Maximum
Age	40	52 (10)	80
Farming experience	2	28 (12)	60

### Assessing urban pearl millet farmers perceptions of climate change

#### Perception of climate change

Perception can be considered as a prerequisite for the adaptation process. In our survey, a majority of the pearl millet farmers (98%) acknowledged the reality and occurrence of climate change, reporting noticeable shifts in climatic patterns over the past 30 years. This heightened awareness could be attributed to Niamey’s status as a commune highly vulnerable to climate change. The high level of awareness is consistent with findings from^18^ in Gambia, Yegbemey et *al. *^14^ in southern Niger, Kutir et al.^3^ in the North Bank Region of the Gambia, and Akponikpè et al.^28^ in Sub-Saharan West Africa. Given Niamey’s high vulnerability to climate change, such awareness is unsurprising. However, while perception is widespread, its depth and alignment with meteorological evidence raise questions about how well subjective awareness matches objective data, a point explored further below.

#### Manifestations of climate change according to urban pearl millet farmers

**Fig. 2:**
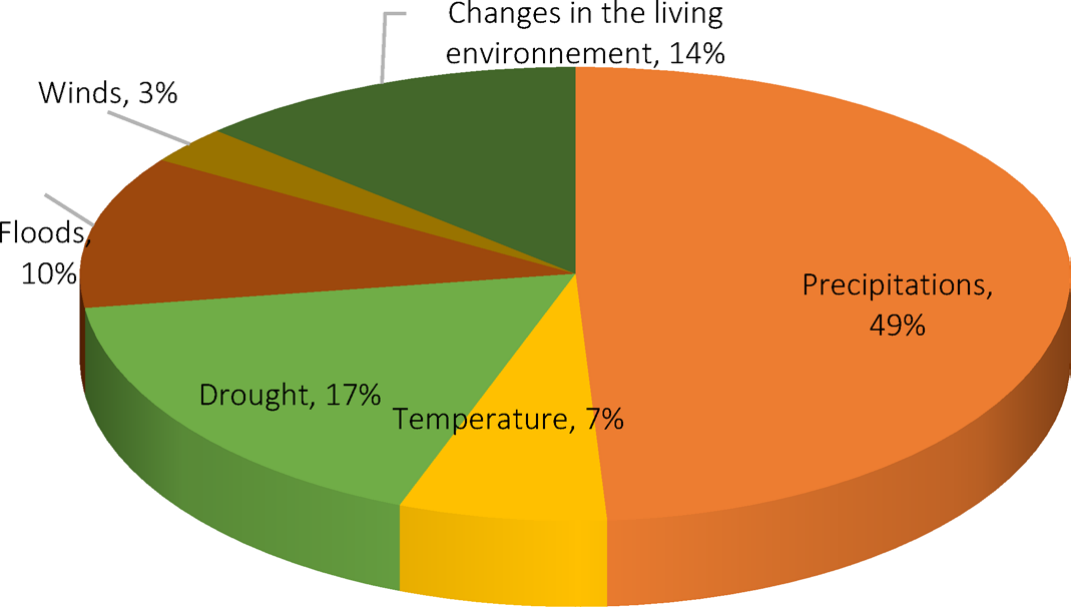
Perceived changes in climate events.

Niamey’s commune V Pearl millet farmers’ perceived climate change primarily through tangible environmental shifts they have experienced over the past 30 years. These include changes in rainfall patterns and seasonality, increased temperatures, more frequent and prolonged droughts, recurrent flooding, and altered wind conditions. Farmers also report ecological impacts such as the disappearance of certain plant and tree species, declining soil fertility, and increased crop failures, factors that have direct implications for food security. These observations are consistent with previous findings by Kutir et al.^3^ and Yegbemey et al.^14^ which emphasized that farmers in Benin and southern Niger often interpret climate change through irregular precipitation, rising temperatures, and environmental degradation. Similarly, Zagre et al.^23^ reported that, in Senegal, smallholders possess a thorough understanding of climate indicators such as annual rainfall patterns, shorter crop seasons, and increasing temperatures, when compared to historical data trends. In our study, 49% of farmers reported a decline in rainfall over the past three decades, along with noticeable disruptions in the timing, frequency, and intensity of rainfall events (Fig. 2). To better understand the depth of these perceptions, farmers were also asked to characterize observed trends across several key climate indicators (increase, decrease, or stability), providing a more structured insight into their climate awareness.

Seventy-four-point seven percent (74.7%) of pearl millet farmers in Niamey’s Commune V reported a decrease in rainfall over the past 30 years, both in terms of quantity and quality of precipitation while 77.3% noted a rise in temperatures, expressed as excessive heat and frequent hot winds. Drought occurrences were perceived as more frequent and prolonged by 80% of respondents, with some reporting dry spells lasting up to seven months. Additionally, 54.7% identified an increase in flood events, particularly linked to recurrent episodes in 2012, 2015, 2018, 2019, 2020, and 2021 (Table 2). These perceptions are not isolated. Similar trends have been documented in other West African contexts, such as among cocoa farmers in Ghana (Buxton et *al*. ,^24^) and diverse crops farmers in Mali^29^, suggesting a shared regional experience of intensifying climate variability. Moreover, the perceptions reported by farmers align closely with meteorological data recorded in Niamey over the same period (see Section 3.2.4).

**Table 2:** Perceived changes in climate occurrences

Climatic occurrences	Frequency of farmers who perceived the changes (%)				
	Increase (%)	Decrease (%)	No change (%)	Don’t know	Total
Rainfall	17 (11.3%)	112 (74.7%)	11 (7.3%)	10 (6.7%)	150 (100%)
Temperatures	116 (77.3%)	19 (12.7%)	11 (7.3%)	4 (2.7%)	150 (100%)
Drought	120 (80%)	22 (14.7%)	6 (4%)	2 (1.3%)	150 (100%)
Floods	82 (54.7%)	52 (34.7%)	11 (7.3%)	5 (3.3%)	150 (100%)

Source: Field survey, 2022.

#### Perceived changes regarding rainfall patterns.

Among all climate-related variables, pearl millet farmers identified rainfall as the one that has undergone the most noticeable and impactful changes. They viewed it as a central element whose disruption has significantly affected the timing and functioning of their agricultural systems. This perception aligns with the findings of Buxton et al.^24^ who reported that cocoa farmers of Ghana similarly regarded rainfall as the most critical and volatile climatic parameter, affecting their cropping activities over time. Moreover, Zagre et al.^23^ further supports this view, revealing that smallholders in Senegal also highlighted the significance of rainfall as a key climatic factor. Their study found that farmers were particularly concerned with seasonal rainfall deficiencies, delayed starts to the growing season, and frequent dry spells, all of which led to decreased yields of both grain and fodder. This shared understanding across regions underscores the central role of rainfall in shaping agricultural practices and adaptation strategies for smallholder farmers. As climate variability continues to intensify, these disruptions are likely to exacerbate challenges in food security, requiring urgent attention to adaptation measures that address the variability of this crucial climatic parameter.

**Fig. 3:**
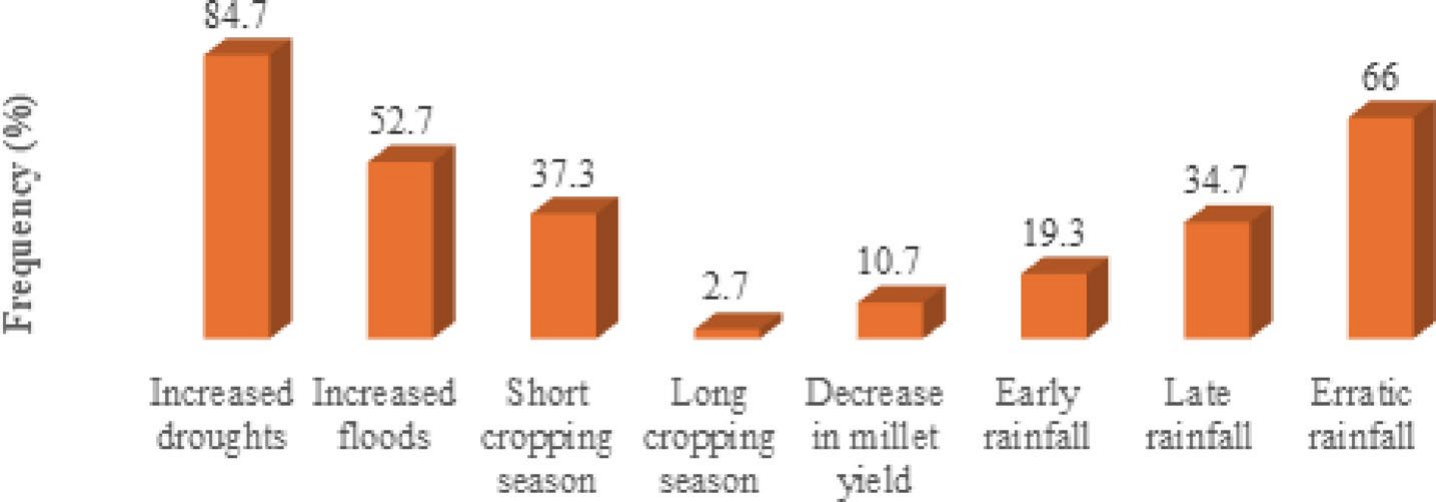
Rainfall trends in Niamey region.

Meteorological observations from Commune V support these perceptions. Over the past 30 years, rainfall trends show a marked decline, accompanied by an increase in dry periods, as reported by 84.7% of pearl millet farmers. These prolonged dry spells have shortened the rainy season and reduced growing windows, directly contributing to lower millet yields for 10.7% of respondents. At the same time, when rains occur, they are sometimes unusually intense, leading to damaging floods (reported by 52.7% of farmers that affect not only crops, but also livestock and human settlements (Fig. 3). Such findings underscore the dual challenge farmers face: managing both insufficient and excessive rainfall, which together heighten production risks and demand increasingly flexible and informed adaptation strategies.

Pearl millet farmers attribute climate change to a variety of causes. For 41.3%, it is seen as an act of divine will, while 40% identify human activities as the main driver. Others cite natural causes (26.7%) or a combination of natural and anthropogenic factors (22%) (Table 3). This diversity of perceptions reflects the coexistence of traditional beliefs and growing awareness of environmental impacts linked to human behavior. The relatively high recognition of anthropogenic causes is notable, as it suggests a potential openness to scientifically grounded adaptation messages*.* Conversely, the predominance of divine attribution among many respondents may influence the degree of agency they feel in responding to climate threats, an aspect that may partially explain variations in adaptation behavior observed in later analysis.

**Table 3:** Perception of Climate Changes causes

	Natural phenomenon	Human activities	God’s decision	Human and nature	Don’t know	Other factors
Rate of perception	26.7	40	41.3	22	5.3	2
Rate of non-perception	73.3	60	58.7	78	94.7	98

Source: Field survey, 2022.

### Assessing meteorological data in Niamey Commune V for the last 30 years (1991 – 2020).

**Fig. 4:**
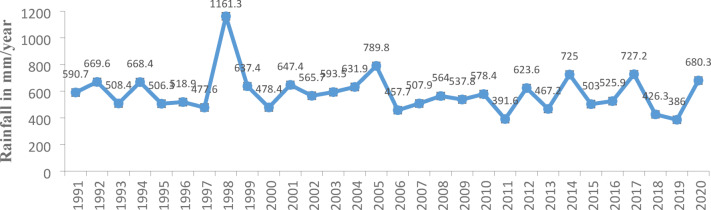
Cumulative rainfall of Niamey V from 1991 to 2020. Source: meteorological department of Niamey Commune V

Pearl millet farmers’ perceptions of climate change are corroborated by meteorological data, which indicated a clear downward trend in annual rainfall and an increase in inter-annual variability over the past 30 years (1991-2020). Data from Niamey station V demonstrate substantial year-to-year fluctuations in rainfall (Fig. 4), with annual totals ranging from 386 mm to 1200 mm. A marked increase was observed between 1991 and 1998, peaking at 1161 mm in 1998, the highest recorded level during the entire period. However, from 1999 onward, rainfall levels declined sharply, dropping to 478.4 mm in 2000 and generally fluctuating within a narrower range of 590-800 mm until 2005. After 1998, annual rainfall never again exceeded 789 mm, marking a shift toward lower and more irregular precipitation. Notably, in 2019, rainfall dropped to 386 mm, the lowest in the entire 30-year series. Despite this apparent downward trend, a Kruskal-Wallis (p=0.533) revealed no statistically significant differences in mean annual precipitation across the three decadal periods. Nevertheless, these figures align with pear millet farmers’ perceptions of declining and erratic rainfall, reinforcing the importance of integrating local knowledge with long-term climate data to better understand climate risks and guide adaptation strategies.

Considering the inter-annual average of the rainfall series (584.91mm), it appears that the decade 1991-2000 appears to be the wettest, while 2011-2020 is the driest among the three decades (Table 4).

**Table 4:** Decadal Rainfall Trends at Niamey Commune V Station

Year	1991-2000	2001-2010	2011-2020
Rainfall (mm)	621.7	587.41	545.61

### Adaptation strategies developed by pearl millet farmers.

#### Local adaptation strategies adopted by pearl millet farmers.

**Fig. 5:**
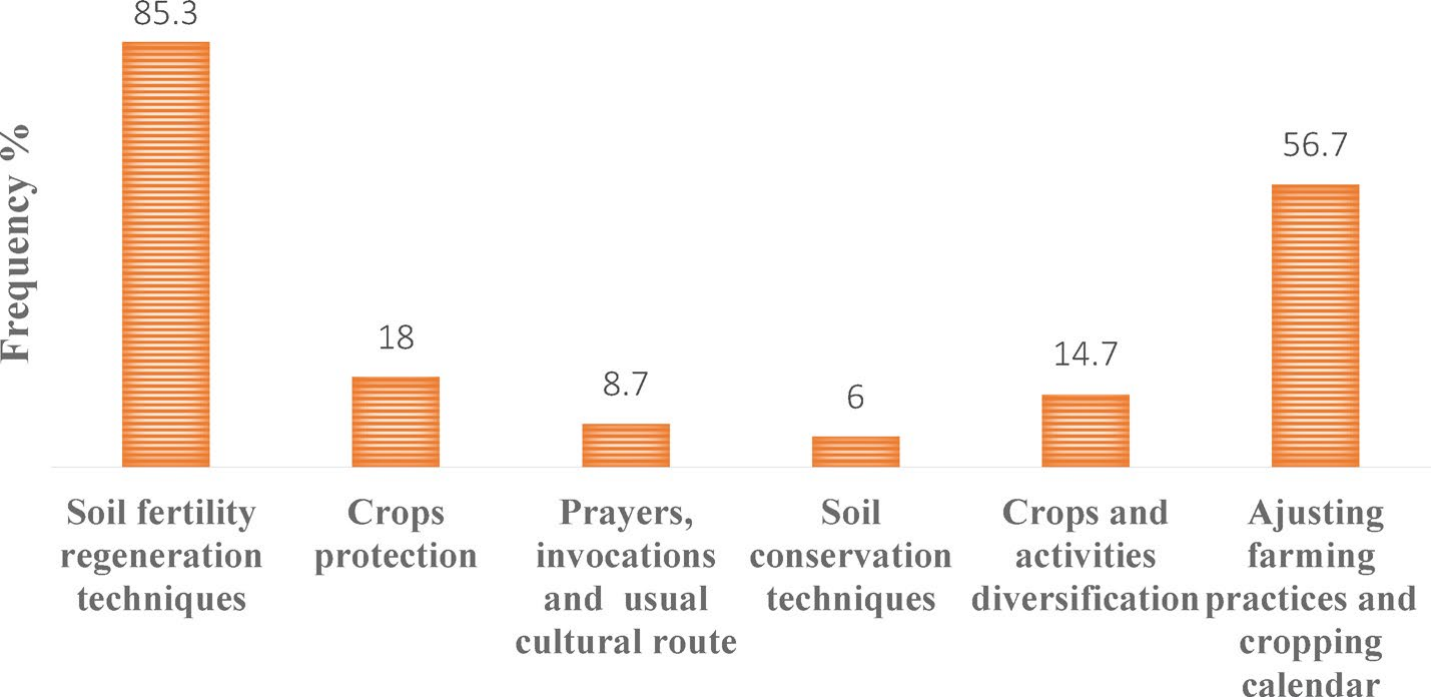
Local coping strategies developed by millet farmers in Niamey commune V.

The majority of pearl millet farmers who acknowledge climate change (98%) have implemented various coping strategies to ensure their crops meet their household needs. These include those developed by farmers based on their local knowledge, as well as those promoted by agricultural services in the commune. Indigenous knowledge-based adaptation strategies have been classified into six categories based on similarities and the resources involved: (1) soil fertility regeneration techniques, (2) soil conservation techniques, (3) cropping practices and agricultural calendar adjustment strategies, (4) crops or activities diversification strategies, (5) crop defense or protection strategies, and (6) other adaptations such as prayers and invocations (Fig. 5).

Among these, the most widely used strategy is soil fertility regeneration (85.3%), which involves using organic manure and/or chemical fertilizers to enhance soil quality. Practices within this category include planting trees between crops, deep plowing to bury manure, and fallowing. Organic manure is typically sourced from oxen or ruminant droppings, or household waste, which is spread over the field. Farmers with oxen park their animals on their land during the dry season to accumulate organic matter, which decomposes into the soil when the rain arrives. Farmers without animals may arrange for relatives to graze animals on their fields to benefit from the manure. Following soil fertility techniques, cropping practices and agricultural calendar adjustments (56.7%) are the most commonly employed strategies. These involve waiting for the onset of rain before sowing, and reseeding if there is a break in the rain that prevents optimal seed germination conditions. Around 18% of farmers use crop protection strategies, including the use of weedicides and pesticides, regular farm maintenance, and the construction of fences to protect crops from animal damage. Approximately 14.7% of farmers adopt diversification strategies, such as crop associations, irrigated cultivation, using improved seeds, millet-bean associations, market gardening, and off-season cultivation. About 8.7% continue traditional practices with invocations and prayers for success. The remaining 6% implement soil conservation techniques, including the alignment of stone blocks to reduce water erosion, as well as Zai and half-moon techniques.

#### Adaptation strategies promoted by extension services.

**Fig. 6:**
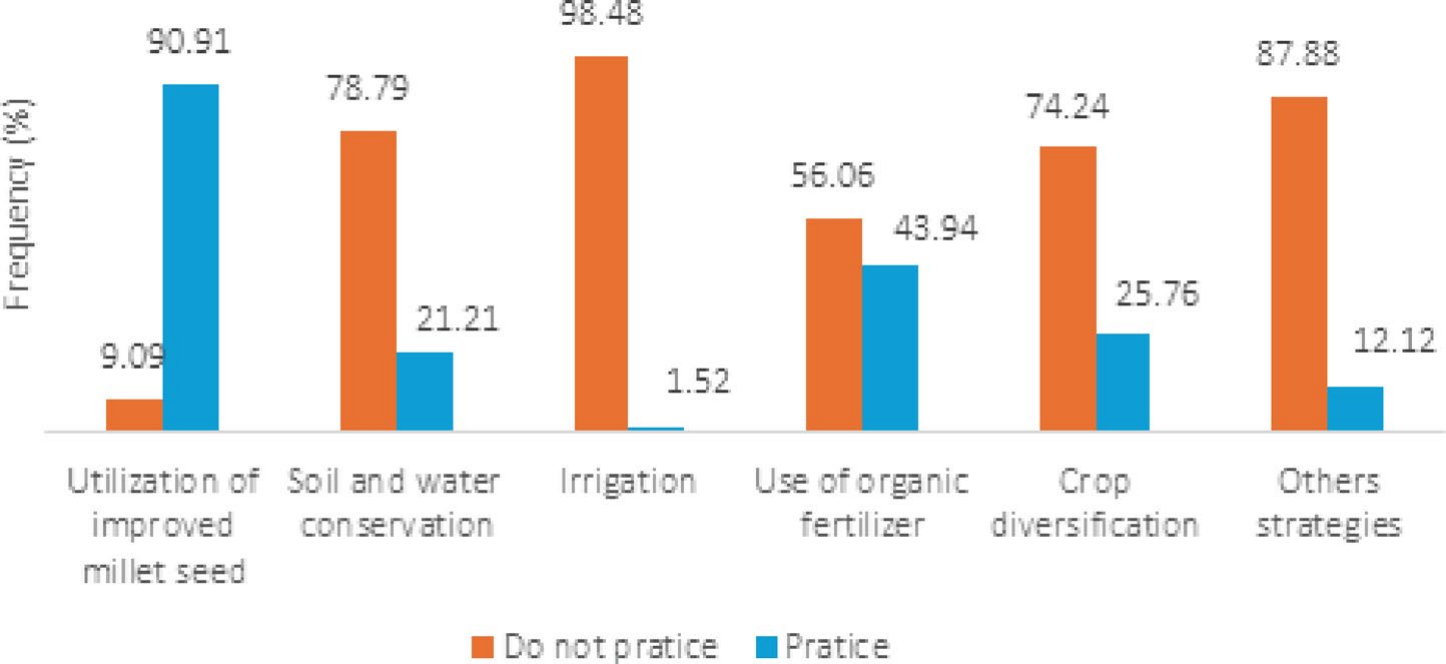
Adaptation strategies learnt from extension services.

Among the 150 pearl millet farmers investigated, sixty-six (44%) reported being engaged with agricultural extension services, which influenced the adaptation strategies they implemented. Figure 6 presents the adaptation strategies implemented by these 66 farmers. Among these strategies, the adoption of improved millet varieties particularly short-cycle types is the most widespread, with 90.9% of these farmers using them. This high adoption rate is largely due to the free distribution of these seeds and their suitability to evolving rainfall pattern (Service agriculture, 2022). Local millet varieties, which require a minimum of three months to mature, are increasingly unsuitable due to the delayed onset and shortened duration of rains. Organic fertilization is the second most adopted practice, used by 43.9% of these farmers, especially those who also raise livestock. They collect animal manure and apply it to their fields before the planting season, allowing it to decompose with the arrival of rain and enhance soil fertility. This method reduces reliance on chemical fertilizers and lowers input costs. While organic fertilization was already practiced locally, extension services have strengthened its adoption by introducing improved composting techniques (e.g., combining crop residues with animal waste) and providing guidance on appropriate application dosages. Other widely promoted practices include crop diversification (25.8%) and water and soil conservation techniques (21.2%). Diversification strategies involve crop associations such as millet-cowpea and cowpea-peanut, off-season cropping, forage cultivation, and small-scale livestock rearing. For soil and water conservation, farmers commonly employ methods like half-moons and the Zaï technique.

The adaptation strategies identified in this study reflect a combination of indigenous knowledge and practices promoted by agricultural extension services. Farmers primarily adopt strategies such as soil fertility regeneration (85.3%), adjustment of cropping calendars (56.7%), use of improved millet varieties (90.91%), crop and activity diversification (40.46%), and water and soil conservation techniques (21.21%). These practices aim to address the major climate induced challenges facing millet production: declining soil fertility, erratic rainfall, pest attacks, and increasing food insecurity. The high adoption rate of improved millet varieties particularly short-cycle and drought-tolerant types demonstrates the effectiveness of input distribution and advisory efforts in enhancing climate resilience. Similarly, training on organic fertilizer production, including the use of crop residues and animal waste, has helped farmers improve composting techniques and optimize nutrient management. Crop diversification and soil and water conservation practices, such as the Zaï and half-moon techniques, are also part of the extension-led toolbox aimed at increasing system resilience.

Indigenous knowledge plays a central role in shaping farmers’ responses to climate variability. Techniques such as soil fertility regeneration through the use of animal manure and compost, cropping calendar adjustments based on observation of seasonal changes, and crop diversification rooted in traditional risk-spreading strategies are all grounded in generations of experience and local ecological understanding. Practices like ritual prayers and invocations also reflect the cultural dimension of resilience, showing how spirituality and belief systems are mobilized alongside technical actions to face climatic uncertainty. These locally rooted strategies demonstrate farmers’ capacity to interpret and respond to their environment using intuitive, context-specific solutions.

The findings in this study are consistent with those observed in other regions of Sub-Saharan Africa, where a combination of traditional and modern adaptation strategies are adopted. For instance, the use of rotation with leguminous crops, shifting planting dates and crop diversification was reported by smallholders of Meouane, Thiel and Daga Birame regions in Senegal^23^. These findings are also consistent with studies conducted in other parts of Sub-Saharan Africa and Central Africa. Okoronkwo et al.^19^ identified prominent traditional adaptation practices aimed at mitigating climate change effects, like the application of organic manure, traditional composting, afforestation, and agroforestry, which are also prominent in this study. Yegbemey et *al*.,^14^ and Takpa et al.^13^ similarly reported the use of both traditional (e.g., sowing adjustments and ritual prayers) and modern practices (e.g., improved seeds and fertilizers) among maize and cotton farmers in northern Benin. In South Kivu, Balasha et *al.,* (2021), noted widespread use of organic amendments, mulching, and crop diversification. Akponikpè et al.^28^ highlighted a growing reliance on soil fertility management and water conservation in Burkina Faso and Niger, whereas Soumana et al.^11^ emphasized the relevance of improved seed varieties and traditional sowing calendars in Niger’s agricultural systems.

### Factors affecting the decision to adapt.

To foster sustainable resilience among farming communities, identifying the determinants of adaptation to climate change is critical. A multinomial logistic regression model was used to analyze the factors influencing the adoption of both indigenous adaptation strategies and those promoted by extension services.

The model estimating the adoption of indigenous strategies was not statistically significant, likely due to homogeneity in characteristics between adopters and non-adopters, combined with a high overall adoption rate (98%) among millet farmers, which limited the variance needed for effective modeling. The multinomial logistic regression model did not reach statistical significance (p = 0.2109 > 0.05; LR (chi^2^ (36) = 42.51); pseudo-R^2^ = 0.1312.

In contrast, the model explaining adoption of strategies promoted by extension services was statistically significant at the 1% level (Prob > chi^2^ = 0.000), with a pseudo-R^2^ of 0.1866, indicating a moderate explanatory power. This indicates that approximately 18.66% of the variation in the adoption of these strategies is explained by the independent variables (Table 5). Among the eight explanatory variables, five were statistically significant: perception of human activities as a cause of climate change, membership in a farmer association, education level, cultivable farmland, and participation in climate adaptation training.

**Table 5:** Result of the logistic regression model

Variables	Coefficient	Standard Error	P>z
Human activities are causing climate change	0.996**	0.397	0.012
Climate information source (Radio)	− 0.051	0.401	0.898
Gender	0.337		0.405
Training in adaptation to climate change	1.708***	0.405	0.000
Membership in an association	1.804**	0.485	0.029
Primary education level	− 1.288**	0.828	0.016
Total area of available land	0.321**	0.534	0.031
Climate information source (Friend)	0.505	0.148	0.199
_cons	− 1.741***	0.393	0.001
Summary of the model	Number of observations = 150 LR chi2(8) =38.40		
	Degree of freedom (ddl)= 8 Prob > chi2=0.0000		
	Log likelihood = -83.687861 Pseudo R^2^ = 0.1866		

^***^: Significant at 1 % (P≤0,01); **: Significant at 5 % (0,01< P≤0,05); *: Significant at 10 % (0,05< P≤0,10).

Gender and the source of climate information had no significant effect on farmers’ adaptation decisions, possibly reflecting uniform access or a lack of actionable support linked to information alone. Our findings on the role of education align with those of Takpa et al.^13^ concerning maize farmers in northern Benin, while the positive influence of association membership contrasts with Soumana et al.^11^ who reported a negative influence in the rural commune of Kourthèye. This divergence may be due to differences in the functionality or inclusivity of farmer organizations across regions. However, our results are consistent with those of Yegbemey et al.^14^ and Avande et al.^30^ suggesting that active and resourceful associations can enhance adaptation. Our findings also resonate with a study by Belle et al.^31^ in Chivi District, Zimbabwe, which emphasized the importance of socio-economic and institutional factors such as marital status, farm size, club membership, and especially access to formal agricultural training in influencing the adoption of climate change adaptation strategies among female-headed households. Their study revealed that while education had an insignificant effect, access to training and strong social networks (e.g., clubs and associations) significantly supported adaptation decisions. These comparative insights reinforce the idea that the success of adaptation strategies depends not only on individual characteristics but also on the availability of inclusive, functional institutions and training systems that can build adaptive capacity at the community level.

#### Perception of human activities as a cause of climate change

The perception that human activities are a major driver of climate change has a positive and statistically significant influence on farmers’ decisions to adopt practices introduced by extension agents at the 5% significance level. This suggests that awareness of anthropogenic climate change enhances receptiveness to external adaptation strategies. This likely reflects a heightened sense of personal responsibility and trust in institutional responses to climate risks.

#### Primary education level

In the reference model, university education serves as the reference category. Farmers with only primary education are significantly less likely (p < 0.05) to adopt adaptation strategies promoted by extension agents. This lower likelihood may stem from a greater resistance to change often associated with lower education levels, a stronger reliance on traditional knowledge systems, and greater influence from social and religious norms that may shape perceptions of formal agricultural innovations.

#### Membership in a farmer association

Farmers who were members of agricultural groups were significantly more likely (p < 0.05) to adopt these strategies. This association may be attributed to the role of associations in disseminating relevant information, sharing best practices, and facilitating access to resources necessary for implementation. Membership often provides additional advantages such as training opportunities, financial support, and access to improved seeds, all of which can enhance farmers’ capacity and willingness to adopt climate adaptation measures.

#### Cultivable farmland

The size of a farmer’s cultivable land has a positive and significant effect (p < 0.05) on the likelihood of adopting extension-promoted adaptation strategies. Farmers with larger landholdings are more inclined to adopt these practices, likely due to greater responsibilities in managing their farms and the need to protect larger investments. They are also more likely to have the financial and material resources required to implement new agricultural techniques.

#### Training in adaptation to climate change

Participation in training sessions on climate change adaptation significantly increases (p < 0.01) the probability of adopting extension-led strategies. Such training enhances farmers’ understanding of climate change, its impacts, and the role of adaptive practices. It also builds confidence in the effectiveness of extension methods, provides practical implementation tools, and encourages collaboration with peers and stakeholders, thereby fostering broader adoption of resilient agricultural practices.

### Smartness of adaptation strategies in terms of adaptation, mitigation, and productivity

This section analyzes the adaptation strategies adopted by pearl millet farmers through the lens of the three pillars of Climate-Smart Agriculture (CSA), as defined by the Food and Agriculture Organization (FAO): adaptation/resilience, productivity, and mitigation. CSA aims to sustainably increase agricultural productivity and incomes, strengthen the resilience of people and food systems to climate change and, reduce or eliminate greenhouse gas emissions when feasible possible^32^.

We evaluated the productivity smartness of each strategy based on its potential to improve food supply, crop yields, and incomes. Adaptation smartness was assessed through water and soil management practices and risk reduction capacity, while mitigation smartness was analyzed through energy efficiency, carbon sequestration, and nutrient management^33–41^. The climate smartness scores of the adaptation strategies are detailed in Table 6 and Table 7.

**Fig. 7:**
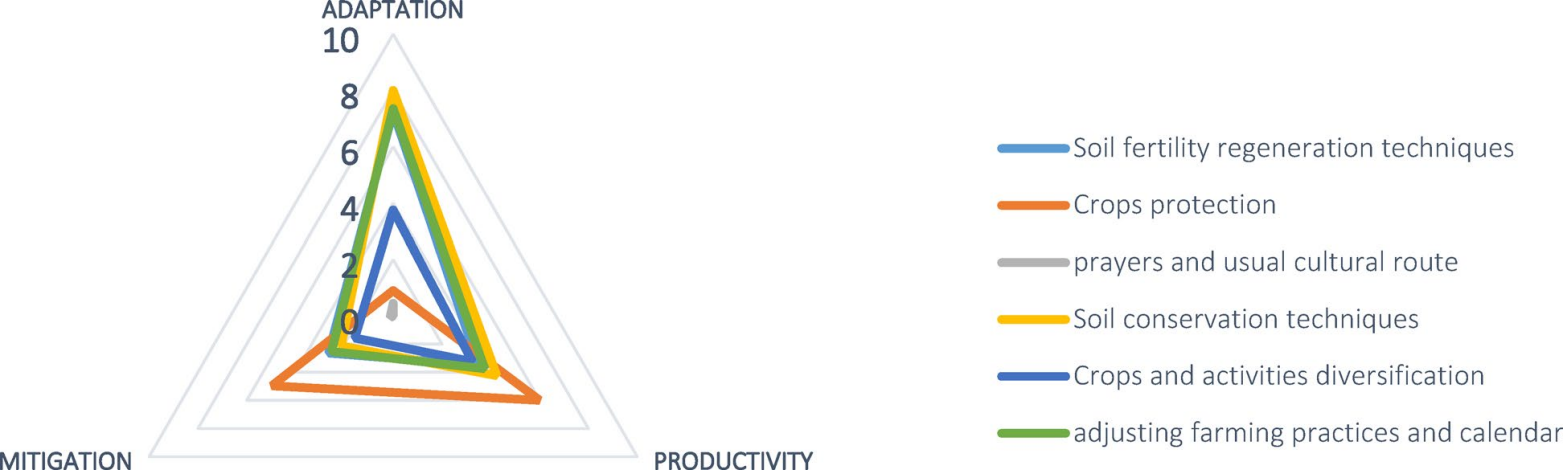
Climate smartness of local adaptation strategies of pearl millet farmers in Niamey Commune V.

Local practices such as soil fertility regeneration (85.3%) and adjusted farming calendars (56.7%) were among the most widely adopted. These practices enhance adaptation by helping farmers optimize planting times based on real-time weather and soil conditions. While fixed planting dates are no longer used, these flexible practices have become crucial in reducing the risks of crop failure. Although less widespread, traditional soil conservation techniques also contribute to adaptation. Conversely, crop protection practices used by farmers tended to focus more on improving productivity than on adaptation or mitigation (soil smartness, water access and risk reduction). In the same way, local methods involving crop diversification or off-farm income-generating activities leaned more toward adaptation by spreading risk rather than increasing yields or reducing emissions. Notably, off-farm diversification emerged as a key strategy to cope with poor harvests Fig. 7).

**Fig. 8:**
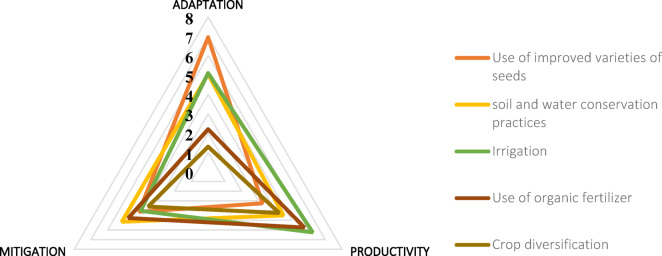
Climate smartness of adaptation strategies from extension officers in Niamey Commune V.

Extension-promoted strategies, such as irrigation, organic fertilizers, and crop diversification, were primarily aligned with productivity goals. Others, such as improved seed varieties and enhanced soil and water conservation practices, were more strongly associated with adaptation. Interestingly, some of these promoted strategies also indirectly support mitigation. For example, improved soil conservation techniques enhance productivity while also increasing carbon sequestration, turning soil into a carbon sink. Likewise, irrigation systems not only address water scarcity (adaptation) and improve yields (productivity) but may also contribute to soil carbon dynamics (mitigation) (Fig. 8).

Agriculture in Africa is primarily driven by the need to increase yields and improve farmers’ incomes. This demand for higher yields is further amplified by the growing global food demand and ongoing hunger crises, yet it is increasingly threatened by the impacts of climate change. Given that the primary goals are to feed the expanding population and increase farmers’ incomes, it is not surprising that farmers prioritize strategies aimed at boosting productivity. This trend is evident in Niamey Commune V, where millet farmers tend to adopt climate-smart agricultural practices that focus heavily on productivity enhancement.

In this context, farmers’ emphasis on productivity influences their choice of adaptation strategies, as they seek ways to adapt to the challenges posed by climate change while maintaining high yields. Practices such as adjusting cropping calendars and engaging in local soil fertility regeneration are commonly employed. These strategies are often seen as means to an end, where the ultimate goal is to increase productivity.

The findings in Fig. 7 suggest that the adaptation strategies employed by pearl millet farmers in Niamey Commune V are less focused on mitigating climate change. This may be due to the fact that the benefits of mitigation are more long-term and less immediately observable compared to those of adaptation and productivity. For many farmers, especially those facing poverty, immediate results are essential. Furthermore, it is likely that the limited attention given to mitigation is linked to a lack of scientific understanding of climate change. While rural farmers are aware that "something has changed" in the climate, evidenced by declining yields, they are often more focused on addressing the immediate need to increase yields rather than tackling the causes of climate change. A typical example of this is the practice of clearing trees and shrubs from fields to create space for planting more crops, which enhances yield but neglects the potential role of trees in climate change mitigation. In contrast, agricultural extension officers promote farming methods that not only align with farmers’ goals of productivity and adaptation but also contribute to mitigation. These officers, likely more familiar with scientific knowledge of climate change, promote improved soil and water conservation techniques that offer dual benefits: they enhance productivity by improving soil conditions and simultaneously act as carbon sinks, contributing to mitigation. Additionally, irrigation systems promoted by extension officers ensure that crops receive sufficient water, which helps farmers adapt to drought while improving plant growth and enhancing the carbon capture potential of the soil. These holistic strategies, as shown in (Fig. 8), integrate the three pillars of climate-smart agriculture: adaptation, productivity, and mitigation unlike the more one-sided approaches observed rather skewed towards one or two pillars as in Fig. 7.

In conclusion, the local adaptation strategies used by pearl millet farmers in Commune V are primarily focused on adapting to climate change impacts and improving productivity, with less emphasis on mitigation. Meanwhile, the strategies promoted by extension officers are more holistic, addressing all three pillars of climate-smart agriculture, adaptation, productivity, and mitigation.

While adaptation and productivity are clearly the most favored components of climate-smart agricultural practices, the mitigation pillar presents a valuable opportunity to combat climate change through agriculture. Future research could explore how to balance the competing interests of the three pillars of climate-smart agriculture to create resilient food systems in the Sahel.

## Conclusion and recommendations

This study shows that pearl millet farmers in Niamey’s Commune V are highly aware of climate change (with 98% of 150 respondents indicating awareness) and, actively adopting measures to cope with its impacts. They perceived climate change through altered rainfall patterns, changes in vegetation, and the increasing frequency of extreme weather events such as floods, strong winds, and heatwaves. Local meteorological data corroborate farmers’ perceptions, indicating a decline in rainfall over the past three decades, which has directly affected millet yields. In response, farmers combine traditional knowledge with practices promoted by extension services. Common adaptation strategies include soil fertility regeneration, adjustments to planting calendars, use of improved millet varieties, crop diversification, and water and soil conservation techniques. Traditional rituals and prayers are also performed to invoke rain or appease spiritual forces. The adoption of extension-driven strategies is positively influenced by several factors: belief in human-induced climate change, membership in farmer associations, education, land availability, and participation in adaptation training. While local strategies mainly focus on improving productivity and building resilience, extension services promote more holistic approaches that address the three pillars of climate-smart agriculture, productivity, adaptation, and mitigation.

This research is based on a relatively small sample size (150 farmers), which may limit the generalizability of its findings. Moreover, data collected through self-reporting could be subject to perception or desirability biases. Future research incorporating in-depth qualitative methods or longitudinal data would enhance the understanding of the long-term effectiveness of adaptation strategies. In this context, this study provides valuable insights into how traditional and modern adaptation practices interact in urban farming contexts. To improve the impact of agricultural climate policies, the following recommendations are proposed:

- Expand access to training by developing targeted training programs on climate-smart agriculture that blend local knowledge with scientific insights.
- Strengthen farmer organizations by investing in inclusive and functional farmer associations to facilitate knowledge exchange and access to resources.
- Promote co-beneficial practices by supporting the adoption of strategies that jointly enhance productivity, resilience, and mitigation such as soil conservation and agroforestry.
- Improve climate information systems through timely, accessible, and actionable climate forecasts to support informed decision-making by farmers.

## Data Availability

The datasets used and/or analysed during the current study are available from the corresponding author on reasonable request.

## Appendix

To enhance transparency and understanding, we have included a separate Excel file containing the CSA evaluation tool developed by CCAFS–CGIAR and applied in this study to assess the climate-smartness of adaptation strategies

See Tables

**Table 6:** Smartness coefficient for local adaptation practices

	Soil fertility regeneration techniques	Crops protection	prayers and usual cultural route	Soil conservation techniques	Crops and activities diversification	adjusting farming practices and calendar
Local adaptation strategies						
Adaptation	7.25	0.9	0.45	8	3.75	7.35
Productivity	3.5	6	0	4.25	3.25	3.75
Mitigation	2.64	4.97	0.13	2.13	1.57	2.53

^6^ and

**Table 7:** Smartness coefficient for adaptation strategies from extension

	Use of improved varieties of seeds	soil and water conservation practices	Irrigation	Use of organic fertilizer	Crop diversification
Adaptation strategies from extension					
Adaptation	6.95	5.05	5.1	2.2	1.3
Productivity	3.25	4.5	6.25	5.75	4.25
Attenuation	4.13	5.16	4.03	4.77	3.59

^7^.
